# A school mental health literacy curriculum resource training approach: effects on Tanzanian teachers’ mental health knowledge, stigma and help-seeking efficacy

**DOI:** 10.1186/s13033-016-0082-6

**Published:** 2016-08-04

**Authors:** Stan Kutcher, Yifeng Wei, Heather Gilberds, Omary Ubuguyu, Tasiana Njau, Adena Brown, Norman Sabuni, Ayoub Magimba, Kevin Perkins

**Affiliations:** 1 Dalhousie University and the Izaak Walton Killam (IWK) Health Centre, 5850 University Avenue, PO Box 9700, Halifax, NS B3K 6R8 Canada; 2 Sun Life Financial Chair in Adolescent Mental Health team, Dalhousie University and IWK Health Centre, Halifax, Canada; 3 Farm Radio International, Ottawa, Canada; 4 Muhimbili National Hospital, Kalenga Street, PO Box 65000, Dar es Salaam, Tanzania; 5 Muhimbili University of Health and Allied Sciences, PO Box 65001, Dar es Salaam, Tanzania; 6 Sun Life Financial Chair in Adolescent Mental Health team, IWK Health Centre, Halifax, Canada; 7 Mental Health and Substance Abuse, Ministry of Health, PO Box 9083, Dar es Salaam, Tanzania; 8 Non Communicable Disease, Ministry of Health, PO Box 9083, Dar es Salaam, Tanzania

**Keywords:** Mental health literacy, School-based intervention, Knowledge, Stigma, Tanzania, Adolescents, Teachers, Mental health, Africa

## Abstract

**Background:**

Mental health literacy (MHL) is foundational for mental health promotion, prevention, stigma reduction, and care; School supported information pertaining to MHL in sub-Saharan Africa is extremely limited, including in Tanzania. Successful application of a school MHL curriculum resource may be an effective way to increase teacher MHL and therefore help to improve mental health outcomes for students.

**Methods:**

Secondary school teachers in Tanzania were trained on *the* African Guide (AG) a school MHL curriculum resource culturally adapted from a Canadian MHL resource (*The Guide*) for use in Africa. Teacher training workshops on the classroom application of the AG were used to evaluate its impact on mental health literacy in a sample of Tanzanian Secondary school teachers. Pre-post training assessment of participant knowledge and attitudes was conducted. Help-seeking efficacy for teachers themselves and their interventions for students, friends, family members and peers were determined.

**Results:**

Paired t test (n = 37) results demonstrate highly significant improvements in teacher’s overall knowledge (*p* < 0.001; *d* = 1.14), including mental health knowledge, (*p* < 0.001; *d* = 1.14) and curriculum specific knowledge (*p* < 0.01; *d* = 0.63). Teachers’ stigma against mental illness decreased significantly following the training (*p* < 0.001; *d* = 0.61). Independent t tests comparing the paired sample against unpaired sample also demonstrated significant differences between the groups for teacher’s overall knowledge (*p* < 0.001). Teachers also reported high rates (greater than ¾ of the sample) of positive help-seeking efficacy for themselves as well as for their students, friends, family members and peers. As a result of the training, the number of students teachers identified for potential mental health care totaled over 200.

**Conclusions:**

These positive results, when taken together with other research, suggest that the use of a classroom-based resource (the AG) that integrates MHL into existing school curriculum through training teachers may be an effective and sustainable way to increase the MHL (improved knowledge, decreased stigma and positive help-seeking efficacy) of teachers in Tanzania. As this study replicated the results of a previous intervention in Malawi, consideration could be given to scaling up this intervention in both countries and applying this resource and approach in other countries in East Africa.

**Electronic supplementary material:**

The online version of this article (doi:10.1186/s13033-016-0082-6) contains supplementary material, which is available to authorized users.

## Background

Mental disorders account for the greatest burden of disease [41, 44] and medical disability [8, 42] for youth worldwide, and the onset of most mental disorders occurs before the age of 25 [11, 32]. However, the majority of youth with mental disorders do not receive adequate care [2, 12, 31, 32, 37, 45]. While the challenges of meeting the mental health care needs of young people are substantial in high-income countries, they are much greater in low-income settings, such as many countries in sub-Saharan Africa (SSA) [4, 30, 31, 47].

Numerous factors contribute to this disparity. For example, Crabb and colleagues [5] reported that there is poor understanding of mental health and mental illness in Africa, and research by other investigators indicates that mental illness is often not generally recognized as such [5, 9]. Furthermore, most countries in SSA have limited mental health resources and services available to meet population mental health care needs [1, 29]. This gap between need and available mental health care resources may be greatest for young people [31]. Compared to high income countries, the negative impact of this reality may be further accentuated as the youth demographic comprises a more substantial proportion of the population distribution across the life span, so that current mental disorders not only are numerically more common but will remain so as this cohort ages over time [3].

The United Republic of Tanzania is a low income country located in the eastern part of SSA [34, 35, 43]. It is currently listed as one of the 49 least developed countries in the world [7]. A majority of the Tanzanian population is youth; those aged between 0 and 14 years comprise almost half of Tanzania’s population (44.34 %) and those aged 15–24 comprise 19.59 % of the population. Combined, those aged between 0 and 24 make up 63.93 % of Tanzania’s population [3]. Historically, mental health care in Tanzania has been provided by a traditional healing system based on the commonly believed association between mental illness and religious and spiritual factors. However, those working as traditional healers are often not regulated by any state governments (country dependant) and do not work within formal health centers [10]. This practice still remains prevalent and the availability of trained mental health professionals is low [47]. Furthermore, traditional healing is more accessible than Western medicine for many people seeking mental health care—especially for those in rural areas due to a lack of available and accessible mental health care professionals, poor transportation and acceptance of spiritual and/or supernatural causes for health problems [33, 36]. The adherence to traditional healing approaches in populations of young people in Tanzania is not known.

Health literacy, which encompasses mental health literacy (MHL), is globally recognized as a foundation for good health and may be a stronger determinant of an individual’s health status than income, employment status, education level, and racial or ethnic group [46]. Our understanding of MHL has changed over the last number of years [10, 16, 18, 21] and is defined as: (1) understanding how to obtain and maintain positive mental health; (2) understanding mental disorders and their treatments; (3) decreasing stigma related to mental disorders; and (4) enhancing help-seeking efficacy (knowing when and where to seek help and developing competencies designed to improve one’s mental health care and self-management capabilities [10, 16, 18]. MHL is considered as the foundation for mental health promotion, early identification of mental illness, intervention and continuing care [20]. However, in Tanzania, there has been little research conducted on approaches to improving MHL. We are only aware of Whyte’s [40] study using a vignette based evaluation framework, which demonstrated that MHL was low and needed improving and reported that Depression was a mental disorder unfamiliar to most people. Furthermore, the majority of respondents who were able to identify Depression believed that it was the caused by supernatural powers [40]. Thus, a need for enhancing MHL in Tanzania exists.

One significant and well-established institution that can be used to address various aspects of mental health (promotion, prevention and care) for young people is the school and, for this reason, school mental health is increasingly receiving global attention [12, 13, 17, 20, 23]. Simply put, schools are an ideal location in which to address MHL for both teachers and students. The cornerstone of school-based MHL initiatives is the improvement of teachers’ MHL. In this manner, teachers not only benefit in terms of developing their own personal MHL competency, but are then also in a position to enhance the MHL of their students [1, 13–16, 20, 38, 39]. One relatively simple method by which this can be achieved is to train teachers in the use of a classroom ready MHL resource and then allow them to use the materials on which they have been trained in their own classes.

The Tanzanian secondary education system has two channels: (1) formal education and (2) non-formal education. The formal education system has 7 years of primary school followed by two cycles of secondary education—a 4-year Ordinary level and a 2-year Advanced level secondary education [28]. Primary education is taught in Kiswahili but secondary education and above is administered in English [28]. Enrollment into Ordinary level is based on satisfactory performance in the National Primary School Leaving Examination whereas enrollment into the Advanced level is based on performance in relevant coursework and meeting the credit requirements during secondary education examinations. Non-formal secondary education is described as organized educational activity outside of the formal education system. It is mainly a continuing (adult) education program for people who did not have the opportunity to attend formal secondary education [27]. In relation to the education system in Tanzania, teachers are described as being essential in promoting quality education; however, overall teaching quality in Tanzania has been reported to be variable by both educational system administrators and teachers themselves [6].

Recent application of such a school-based approach in Canada that integrates MHL into school curriculum by teaching teachers how to apply a modular, freely available, on-line curriculum resource [22 has demonstrated significant and sustained improvements in knowledge and reduction in stigmatizing attitudes for both teachers and students in a number of Canadian studies [14, 19, 24–26]. A recent application of this approach in Malawi [17], using a culturally-adapted version of this resource [the African Guide (AG)], has demonstrated similar positive outcomes in teachers to those obtained in Canadian studies.

This study is similar to the one conducted in Malawi and serves to investigate the impact of implementing a culturally adapted version of the classroom ready MHL resource in Tanzania, developed and applied as part of a Grand Challenges Canada funded project addressing youth Depression in Malawi and Tanzania [22]. The Guide, which had been previously adapted for use in Malawi was further revised for application in Tanzania by a working group of Tanzanian mental health experts who included a psychiatrist, a psychologist and a social worker. Their revisions were incorporated into the material and this product was then identified as the African Guide. It was this version that was applied in Tanzania. In the current study, teachers who received earlier training on the use of the Guide returned for a refresher workshop. This was deemed necessary by the field project team, as teachers who were previously trained on the resource requested further training. Prior to the outset of this study, the research team hypothesized that this additional training intervention would significantly and substantially improve mental health knowledge and decrease stigma of teachers participating in this refresher course.

## Methods

The curriculum resource, which had been previously adapted for use in Malawi was further revised for application in Tanzania by a working group of Tanzanian mental health experts who included a psychiatrist, a psychologist and a social worker. Their revisions were incorporated into the material and this product was then identified as the African Guide. It was this version that was applied in Tanzania. This material was then translated into Kiswahili by the expert team, lead by two of the authors (OU and TN). The AG consists of a teachers’ mental health knowledge self-study study guide, a self-evaluation test, and six classroom ready modules. The six modules are: (1) the stigma of mental illness; (2) understanding mental health and wellness; (3) information about specific mental illnesses; (4) experiences of mental illness; (5) seeking help and finding support; and (6) the importance of positive mental health. All modules include learning objectives, major concepts addressed, lesson plans, classroom activities and teaching resources (the Canadian version is available at http://www.teenmentalhealth.org).

### Sampling

A Master Trainers Team (MTT) comprised of four mental health experts—two psychiatrists and two psychologists—were trained over a period of 2 days on the use of the African Guide by its lead developer (SK). MTT members then trained teachers who were purposefully selected by education administrative authorities from 35 secondary schools in Arusha District Council and Meru District Council, in the classroom application of the AG. Following field application of the AG, teachers requested a training upgrade session. Seventy-five teachers were available to participate in this refresher training intervention, however only 61 completed the full 3 days. Teachers recruited for the training program taught both ordinary and advanced levels.

### Study design

In order to evaluate the impact of participating in the refresher training exercise on the mental health knowledge and attitudes of teachers, a pre- and post-study design was used. Participants’ knowledge about and attitudes towards mental health and mental illness were measured at the beginning and again at the completion of the 3-day refresher training, using the same knowledge and attitudes assessment tool as was used in Malawi. To assure anonymity, participants were asked not to provide any identifying information on the test materials and anonymous identifiers such as month of birth, postal code and mother’s first name were used to link participants’ pre- and post-training responses. The initial training was conducted and data obtained in the fall of 2014 (see Additional file 1 for questionnaire); refresher training was conducted and data obtained in the spring of 2015. Ethics approval for research in Tanzania was received from the National Institute for Medical Research (NIMR) in Tanzania. Permission to conduct the intervention was obtained from the Regional Education Officer and the Regional Medical officer respectively.

### Procedures

The classroom MHL resource training intervention for teachers was applied in the Arusha and Meru Districts of Arusha Region of Tanzania in the summer of 2015. This report addresses the application of refresher training provided to teachers who had previously undergone a 3 day session in the classroom application of the AG. Sixty-one teachers from that original group also participated in and completed this refresher training intervention, occurring approximately 6 months later. Since, due to logistical challenges experienced by the Tanzanian field research team the data from the original training session is not available, this study reports only on the outcomes of the refresher training.

Prior to the initial training session, youth mental health services were not available at community health facilities and referrals were therefore unlikely to result in receiving appropriate care. In the period of time between initial and refresher training, a small cadre of community health care providers received training in the diagnosis and treatment of adolescent mental disorders with a particular emphasis on Depression. For this reason, the refresher training also assessed teacher referrals of students to community health centers for mental health related problems in the period between the initial and refresher training sessions. Prior to the provision of the initial teacher’s training program and the training of local community health care providers, teachers had not suggested that any student seek mental health care even when they suspected them to have a certain mental health problem.

### Questionnaire and outcome measures

An evaluation of mental health knowledge and attitudes was conducted using previously applied [15] pre- and post-tests that were reviewed for cultural appropriateness by Tanzanian mental health experts. Teachers were also asked questions about their identification and referral of students and others for mental health care 6 months following their participation in the initial training session. Thirty-eight fully completed pre- and post-test pairs were matched. Tests which were not fully completed were not matched for paired “t test” analysis. The knowledge component of the tests consist of 30 questions (Cronbach’s α pre-test = 0.601; Cronbach’s α post-test = 0.435)—the first 22 questions assess general mental health knowledge and the remaining 8 questions are directly related to the content in the AG resource. Possible responses are true’, ‘false’ and ‘I don’t know’’. Participants were instructed to choose only one option per question and were encouraged to mark ‘I don’t know’ rather than guessing.

Eight questions (Cronbach’s α pre-test = 0.661; Cronbach’s α post-test = 0.631) were used to measure attitudinal change using a seven-point Likert Scale, ranging from ‘strongly disagree’ to ‘strongly agree’. A total positive attitude score out of 56 was calculated. Examples of attitudinal questions are “most people who have a mental illness are dangerous and violent”, “mental illness is usually a consequence of bad parenting or poor family environment” and “people who are mentally ill do not get better”.

Three additional questions (Cronbach’s α pre-test = 0.667; Cronbach’s α post-test = 0.748) assessed participants’ comfort levels in regards to addressing the mental health needs of students using a five-point Likert Scale, ranging from ‘strongly disagree’ to ‘strongly agree’. A total comfortability score out of 15 maximum points was calculated.

Participants also responded to six questions regarding whether they had identified students/friends/family who may have a mental health problem, advised students/friends/family to seek professional help for a mental health problem and if they recognized and/or sought professional help for a personal mental health problem. Questions were in a ‘yes’ or ‘no’ format. If participants selected ‘yes’ for a particular question, they were asked to identify how many as either ‘1–5’, ‘6–9’ or ‘10+’ people.

### Analysis

Using anonymous identifiers, a total of 38 pre and posts tests were matched for analysis, using paired t tests, from the refresher training session. As nearly half of the data were not matched, we also conducted independent t tests, treating participants who completed both pre and post-tests (n = 38) with participants whose questionnaires were not matched (n = 24) as two independent groups to compare against their results (post-test of the matched groups against data from the unmatched group). The independent analysis was conducted to determine if the results from training could only be noticed in those who had matched assessments or if differences between pre- and post-tests were a result of the training alone, regardless if the tests could be matched or not.

IBM SPSS statistics 22 was used to conduct paired and independent *t* tests to evaluate the differences in knowledge, attitude scores at pre-test and immediately following the 3-day refresher training. Cohen’s *d* was calculated by dividing the mean difference of the paired groups by the pooled standard deviation. Descriptive statistics were also used to provide information regarding the number of teachers who identified students at the risk of having a mental health problem as well as the number of teachers who advised students to see a mental health professional about their mental health problems.

## Results

### Participant characteristics

A total of 61 Tanzanian secondary school teachers began the refresher training on the African Guide. Twenty-nine males (47.5 %) and 29 females (47.5 %) participated in the training; three participants did not provide information on sex (4.9 %). Of the 61 total teachers, 38 (15 females, 23 males) participants had knowledge and attitudes pre- and post-tests that could be matched for statistical analyses. Teachers taught a wide variety of subjects: language arts (English, Kiswahili); finance (book-keeping, commerce); industry (information and communication technology/ICT); social studies (geography, history, bible knowledge, civics, mental health); and sciences (biology, chemistry, physics). A majority of participants taught social studies (n = 21, 34.4 %), followed by sciences (n = 12, 19.7 %), language arts (n = 6, 9.8 %), other (n = 5, 8.2 %), finance (n = 4, 6.6 %), and industry (n = 1, 1.6 %); 12 participants (19.7 %) did not provide this information.

### Knowledge results

Outcomes of the knowledge assessment survey were completed in three separate analyses: (1) mental health knowledge, (2) curriculum resource specific knowledge and (3) overall knowledge score combining scores from (1) and (2). The mental health knowledge score, at baseline, ranged from 9 to 16 out of a possible 22. The average mental health knowledge score was 14.3 (65 %) with the median being 14 (64 %). The curriculum resource specific knowledge score, at baseline, ranged from 0 to 8 out of a possible 8. The average curriculum specific knowledge score was 5.36 (67 %) with the median being 6 (75 %). The overall knowledge score, at baseline, ranged from 10 to 26 out of a possible 30. The average overall knowledge score was 19.6 out of 30 (65 %) with the median being 20 (67 %).

#### Mental health knowledge

For matched assessments, outcomes of the mental health specific questions on the out of a possible score of 22, the pre-training average was 14.16 (SD ± 2.19) and the post-training average was 16.68 (SD ± 2.23). This difference is statistically significant; *t*(37) = 5.46, *p* < 0.001; Cohen’s *d* = 1.14 (see Table 1). The effect size for this analysis exceeds Cohen’s condition for a large effect (*d* = 0.80).

**Table 1:** Effect of AG curriculum resource training on teachers’ scores at pre- and post-training, n = 38

	Pre-training	Post-training	Cohen’s *d*	*p* value
Knowledge				
Mental health specific	14.16 (2.18)	16.68 (2.23)	1.14	***
Curriculum specific	5.61 (2.01)	6.66 (1.24)	0.63	**
Overall	19.76 (3.57)	23.34 (2.63)	1.14	***
Attitudes				
Towards mental health	41.39 (8.38)	46.08 (7.02)	0.61	***
Comfort				
Addressing mental health needs	13.25 (1.86)	13.72 (1.34)	0.19	>0.05

Data are mean (SD)

^*^ *p* value <0.05

^**^ *p* value <0.01

^***^ *p* value <0.001

#### Curriculum specific knowledge

For matched assessments outcomes of the curriculum specific questions on the knowledge assessment survey found that, out of a possible score of 8, the pre-training average was 5.61 (SD ± 2.01) and the post-training average was 6.66 (SD ± 1.24), a statistically significant difference; *t*(37) = 3.06, *p* < 0.01; Cohen’s *d* = 0.63 (see Table 1). The effect size for this analysis meets Cohen’s condition for a moderate effect (*d* = 0.50).

#### Overall knowledge score

For matched assessments outcomes of the total knowledge assessment survey found that prior to the training, out of a possible score of 30, the pre-training average was 19.76 (SD ± 3.57) and the post-training average was 23.34 (SD ± 2.63), a statistically significant difference; *t*(37) = 5.61, *p* < 0.001; Cohen’s *d* = 1.14 (see Table 1). The effect size for this analysis exceeds Cohen’s condition for a large effect (*d* = 0.80). Using an independent samples t test to compare results of participants who were matched (n = 38, M = 23.34, SD ± 2.63) against those who were not matched (n = 24, M = 19.25, SD ± 4.01), we found a statistically significant difference: *t*(35.57) = 4.43, *p* < 0.001.

#### Attitude results

The attitudes assessment assessed stigma related to mental health. The overall positive attitude score, at baseline, ranged from 5 to 44 out of a possible 56. The average was 30.9 and the median was 31 falling into the “not sure” scoring category. The question in which teachers had the highest negative attitude scores (“strongly disagree” and “disagree” categories represent over 75 % of responses) was questions 6. Questions were reverse scored where necessary.

#### Attitude scores

For matched assessments outcomes of teachers’ attitudes towards mental illness found that prior to training, out of a possible score of 56, the pre-training average was 41.39 (SD ± 8.38) and the post-training average was 46.08 (SD ± 7.02). These results were statistically significant, *t*(37) = 4.95, *p* < 0.001; Cohen’s *d* = 0.61 (see Table 1). The effect size for this analysis meets Cohen’s condition for a moderate effect (*d* = 0.50). Using an independent samples t-test to compare results of participants who were matched (n = 38, M = 46.08, SD ± 7.02) against those who were not matched (n = 24, M = 31.92, SD ± 5.25), we found a statistically significant difference: *t*(58.15) = 9.06, *p* < 0.001.

#### Comfort results

The comfort score assessed teachers’ improvements in relation to talking to, identifying and/or advising students with mental health problems/concerns. Comfort score, at baseline, ranged from 0 to 15 out of a possible 15. The average was 12.6 and the median was 14 falling into the “agree” and “strongly agree” categories, respectively.

#### Comfort score

For matched assessments there was no significant difference in participants’ self-reported comfort in addressing youth mental health needs from pre-training (*M* = 13.25, SD ± 1.86) to post-training (*M* = 13.72, SD ± 1.34); *t*(35) = 1.45, *p* > 0.05; Cohen’s *d* = 0.19 (see Table 1). The effect size for this analysis meets Cohen’s condition for a small effect (*d* = 0.29). Using an independent samples t-test to compare results of participants who were matched (n = 38, M = 13.71, SD ± 1.35) against those who were matched (n = 24, M = 12.54, SD ± 3.55), we found no statistically significant difference between samples: *t*(27.28) = 1.54, *p* > 0.05.

### Referrals for mental health care since the initial training program

**Fig. 1:**
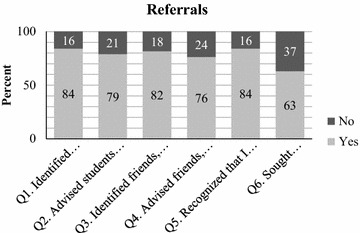
Percent of ‘*Yes*’ to ‘*No*’ responses for referrals. This *figure* shows percentage of yes to no responses by educators for each question asked as it relates to identifying and advising students to seek mental health services as well as recognizing if educators themselves feel they may have a mental health concern and whether they sought help

**Fig. 2:**
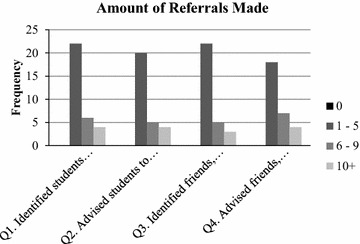
Amount of people referred to seek professional help. This *figure* shows the amount of students, friends, family members, and/or co-workers that were identified as having a mental health problem and were advised to seek professional help. *Frequency* represents how many educators identified/advised 0 people, 1–5 people, 6–9 people, and 10+ people

A majority of participants indicated that, since their refresher training session, they identified and/or advised students, friends, peers, or family members to seek professional help for a mental health problem or sought professional help themselves (see Figs. 1, 2). Over half of the teachers (63 %) noted that they had personally sought help for a mental disorder or mental health problem since the initial training period. Over ¾ of the cohort (84 %) reported that they had identified students who had a mental disorder or mental health problem. The number of students teachers identified for mental health care totaled over 200. Furthermore, In addition, over ¾ of teachers (82 %) reported that they identified friends, peers or family members whom they thought may have a mental disorder or mental health problem and over ¾ of teachers (76 %) advised that they sought professional help.

## Discussion

This is, to our knowledge, the first study reporting on the successful impact of a school based mental health literacy resource training program on mental health knowledge enhancement, stigma reduction and help-seeking efficacy (for self, students and others) in the country of Tanzania. Simply put, this teacher mental health literacy resource (the AG) refresher-training program demonstrated significant and substantial improvements in teacher’s mental health knowledge and significant and substantial decreases in teacher’s stigma. These results are congruent with the findings from the study conducted in Malawi [17] as well as in Canada [14, 22] demonstrating the use of The Guide (both the original and modified version) as being effective tools for increasing mental health literacy within the school system on an international level. These findings support the need for mental health literacy refresher training for teachers as this cohort had previously been exposed to a similarly focused training experience.

Additionally, these results demonstrated that the initial training experience may have produced a behavioral impact related to mental health help seeking efficacy on this teacher cohort. These results demonstrated high levels of teacher’s help-seeking efficacy during the training interregnum period, including obtaining help for themselves and also suggesting students, friends, family members or peers obtain professional mental health care. Comfort levels in helping student’s address their mental health needs were high at baseline and did not change significantly as a result of the intervention. These findings suggest that once teachers receive training in how to apply a mental health literacy resource (the AG) in their classrooms, they spontaneously use their new knowledge to reach out to and assist their students, friends, family members and peers. To our knowledge, this finding of a positive behavioral outcome resulting from simply learning how to teach a classroom mental health literacy resource (the AG) is the first such report, not only in Tanzania but globally. This suggests that a pedagogically familiar classroom based mental health literacy resource taught to teachers can, in and of itself enhance identification and encourage help-seeking behaviors directed to access mental health care. Should this finding be replicated in further research it will provide support for the importance of a teacher based classroom mental health literacy resource intervention in enhancing self, student and others direction towards mental health care.

Our independent t test demonstrated that there was a significant difference between participants who were matched and those who were not matched on the knowledge and attitudes portion of the guide but no significant difference was found for the comfort score results. This indicates that our findings were unlikely to be biased and may be considered to be representative of the Guide impact on this cohort of teachers since the two results are congruent.

## Limitations

Although this study demonstrates success in improving teachers’ mental health literacy by enhancing knowledge and decreasing stigma, it has certain limitations. While it is the first of its kind in Tanzania, it was a cohort study and not a controlled trial with a relatively modest sample size. Thus, although it is unlikely (given the pre-post study design over a short time period) that these results would have occurred without the intervention, a controlled study would be preferred. Furthermore, although the sample size is the largest ever reported on in the Tanzanian context and teachers were selected across a wide spectrum of school types and locations, it may be that the study participants are not completely representative of the Tanzanian teacher population as a whole and thus replication studies are needed. Further, as this report addressed a refresher study, it would be useful to follow a cohort of teachers, to determine whether the change will be maintained overtime.

This study also did not assess how many teachers who self-identified need for a mental health assessment or how many students, friends, family members and peers that teachers had suggested obtain a mental health assessment actually proceeded to obtain one. Nor did this study examine the ability of those so counseled to access an appropriate or timely mental health assessment nor determine how many received mental health care. These important considerations however lie outside of this study and the findings of teacher’s behavioral change related to improved mental health seeking efficacy are nonetheless encouraging. The impact, if any, of the development of community based mental health care capacity where none had previously existed, concurrently with this intervention, had on these findings is also not known. Future research addressing these important issues already underway or will be conducted as part of a series of follow-up studies.

An additional possible limitation is that given the design of the project we are not able to determine what the proportional impact the teacher training and application of the AG, compared to the newly available youth mental health care capacity as a result of the training of community health care providers, had on referrals of students for treatment. Ethically it is not possible to train teachers and provide information to students about mental disorders and not have any services available to meet identified needs. However, once the services are well established additional research may be able to determine what proportion of referrals comes from schools compared to other referral sources.

One remaining limitation with this study is the lack of data from the initial training session that was not available for comparison with the refresher training data. This was due to a misunderstanding of the field research team as to which evaluation tools were to be used in the initial training session, and the tool that was used was not the correct one. Such real-life difficulties are a reality in health care research conducted in low-income settings and illustrate a field research challenge that will be mitigated in future research by more rigorous monitoring processes and procedures.

## Conclusion

These results described here are similar to those reported in a previous study conducted in Malawi, using a comparable design and similar evaluation tools [17]. Similar results have also been reported from studies conducted in Canada, a very different location from Malawi or Tanzania [14, 24]. Since this approach to school mental health literacy is relatively easy to apply, does not depend on fidelity of application, is consistent with usual and well established pedagogical approaches, builds on existing education systems by enhancing the competencies of available human resources (and thus is inherently sustainable) and has demonstrated replicable results across varied jurisdictions, it may be possible to consider its application in other African countries where there is demonstrated interest in enhancing school mental health activities and improving mental health outcomes for youth and teachers alike. Currently, this curriculum resource training program has been ongoing under the supervision of related government agencies in project participating locations in both Tanzania and Malawi after the initial intervention was completed. Finally, consideration to implementing this material into pre-service teacher training is necessary if long-term stability of enhancing teacher MHL is to be realized.

## Additional file

10.1186/s13033-016-0082-6 The Questionnaire assessed teachers’ knowledge, attitudes and confidence regarding mental health; the amount of students, friends, peers, and family members teachers identified as having a mental health problem and advised to seek help; and their self-identification of a mental health problem and help-seeking intentions.
