# Supplementary material for: A school mental health literacy curriculum resource training approach: effects on Tanzanian teachers’ mental health knowledge, stigma and help-seeking efficacy
Source: Int J Ment Health Syst. 2016 Aug 4;10:50. doi: 10.1186/s13033-016-0082-6 (PMC4973111; doi:10.1186/s13033-016-0082-6)
Supplement: Supplementary file 1 — 10.1186/s13033-016-0082-6 The Questionnaire assessed teachers’ knowledge, attitudes and confidence regarding mental health; the amount of students, friends, peers, and family members teachers identified as having a mental health problem and advised to seek help; and their self-identification of a mental health problem and help-seeking intentions. [file 13033_2016_82_MOESM1_ESM.pdf]

School Mental Health &

THE AFRICAN SCHOOL MENTAL HEALTH CURRICULUM

(EDUCATOR PRE)

This survey is designed to assess the baseline knowledge regarding school mental health and the African School Mental Health Curriculum Guide.

To help us match your anonymous responses between surveys done at the start and end of the course please tell us: a) The **month** you were born__________, b) Your post code____________
c) Your **mother's** first name______________________________

In what school do you teach?___________________________ Gender: Male ☐ Female ☐

What grade do you teach? 9 ☐ 10 ☐ 11 ☐ 12 ☐ other ______________________

What subject(s)/course(s) do you teach?________________________,

Section A: For each of the following statements select True, False, or Do Not Know by marking an **X** in the appropriate box.

Question	True	False	Do Not Know
1. Mental health and mental illness both involve the brain and how it functions.			
2. People who have mental illness can at the same time have mental health.			
3. The brain can affect the way the body functions but the body can not affect the way the brain functions.			
4. The frontal lobes of a young person's brain continue to grow and develop until about the age of 25 years.			
5. Three of the functions of the brain include thinking, signaling and behavior.			
6. Every person's mood can fluctuate up and down naturally.			
7. The brain acts to help control the functioning of the heart, lungs, and fingers.			
8. Both genetic problems and infections can cause the brain to get sick and stop functioning normally.			
9. A person can have a mental illness without feeling stressed out.			
10. People who have a mental illness are frequently violent.			
11. Most people who have a mental illness get well and stay well with treatment.			
12. Vitamins and meditation are good treatments for most mental illnesses.			
13. People who have schizophrenia often get a split personality.			
14. Depression and Bipolar Disorder are tow examples of the type of mental illnesses called mood disorders.			

Question	True	False	Do Not Know
15. An anxiety disorder happens when a person's brain detects the presence of danger - such as a dog attacking.			
16. Panic Disorder commonly beings in adolescence.			
17. A panic attack comes on suddenly and typically lasts one or more days.			
18. Attention Deficit Hyperactivity Disorder has three components including attention problems, hyperactivity, and depression.			
19. Suicide in young people is mostly related to bullying and has little to do with mental illness.			
20. Self-harming behaviors may accidentally lead to death.			
21. Treatment of mental disorders has three purposes including, relieving symptoms, restoring functioning, and promoting recovery.			
22. The website www.teenmentalhealth.org is a good place for young people to get information about mental health and mental illness.			
23. When learning how mental disorders are classified into categories (e.g., ADHD, Depression, Anxiety etc) it is important to first learn about the connection between brain functions and mental disorders.			
24. The *Mental Health & High School Curriculum Guide* developed in partnership with the Sun Life Chair in Adolescent Mental Health, is a mental health literacy program designed to assist teachers in educating students about mental health.			
25. The *Mental Health & High School Curriculum Guide* consists of 6 modules that must be taught in a specific order.			
26. The *Mental Health & High School Curriculum Guide* can be integrated into preexisting teacher plans or can be taught separately in a blocked chunk of time, all depending on the school needs.			
27. The *Mental Health & High School Curriculum Guide* contains a series of classroom activities, such as, two slide shows and a video relating to young peoples' experiences with mental illness.			
28. The purpose of the *Mental Health & High School Curriculum Guide* is to promote students' understanding of mental illness			
29. *The Mental Health & High School Curriculum Guide* does not address the importance of positive mental health and only focuses on mental illness.			
30. The *Mental Health & High School Curriculum Guide* is designed specifically for use in Grades 9 but can be adapted for other high school grade levels.			

Section B: This section of the survey is designed to find out about your attitudes toward the statement.

For each of the following statements please mark an **X** in the box that you feel best describes your attitude toward the statement. Please select only one answer for each statement.

	Strongly Disagree	Disagree	Disagree a little	Not sure	Agree a little	Agree	Strongly Agree
1. It is easy to tell when someone has a mental illness because they usually act in a strange or bizarre way							
2. A mentally ill person should not be able to vote in an election							
3. Most people who have a mental illness are dangerous and violent							
4. Most people with a mental illness can have a good job and successful and fulfilling life							
5. I would be willing to have a person with a mental illness at my school							
6. I would be happy to have a person with a mental illness become a close friend							
7. Mental illness is usually a consequence of bad parenting or poor family environment							
8. People who are mentally ill do not get better							

Section C

Please complete the following questionnaire according to the scale: 1 – strongly disagree; 2 – disagree; 3 – neutral; 4 – agree; 5 – strongly agree

As a result of the training I received in mental health, I am better able to:

1. Talk to students about their mental health concerns

   1 ☐ 2 ☐ 3 ☐ 4 ☐ 5 ☐

2. Identify students who may have mental health problems or concerns

   1 ☐ 2 ☐ 3 ☐ 4 ☐ 5 ☐

3. Advise students where they can go to get help if they have concern about a mental health problem or disorder

   1 ☐ 2 ☐ 3 ☐ 4 ☐ 5 ☐

Section 2

Since the training I received in mental health, I have:

1. Identified students who may have a mental health disorder or mental health problem Yes_________ No_________

If yes, how many?

1 – 5 ☐ 6 - 9 ☐ 10+ ☐

2. Advised students to seek professional help for a mental disorder or mental health problem? Yes_________ No_________

1 – 5 ☐ 6 - 9 ☐ 10+ ☐

3. Identified friends, peers or family members who may have a mental health problem Yes______ No_______

If yes, how many?

1 – 5 ☐ 6 - 9 ☐ 10+ ☐

4. Advised friends, peers or family members to seek professional help for a mental health problem or mental disorder? Yes_____ No______

If yes, how many?

1 – 5 ☐ 6 - 9 ☐ 10+ ☐

5. Recognized that I myself may have a mental disorder or mental health problem? Yes______ No_____

6. Sought professional health myself for a mental health problem or mental disorder Yes______ No ______
